# Supplementary figures and images for: Kv3 Channels Contribute to the Excitability of Subpopulations of Spinal Cord Neurons in Lamina VII
Source: eNeuro. 2022 Feb 17;9(1):ENEURO.0510-21.2021. doi: 10.1523/ENEURO.0510-21.2021 (PMC8868027; doi:10.1523/ENEURO.0510-21.2021)

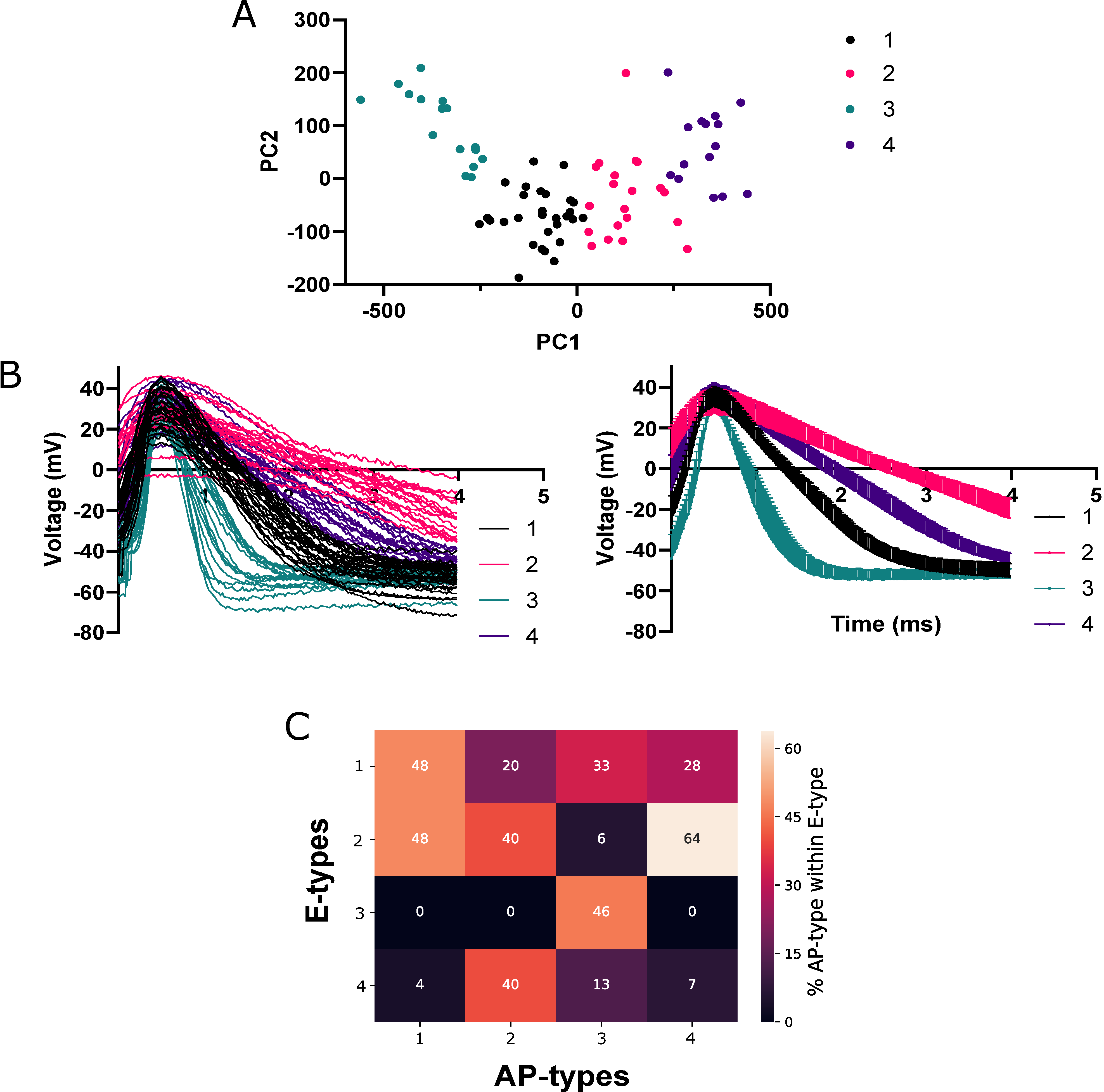

Supplement: Extended Data Figure 2-1 — Correlation of AP waveform with e-types. A, The first AP waveform produced at rheobase for each neuron was clustered. Scatterplot of principal components 1 and 2 indicating summarizing clusters. B, Individual (left panel) and average waveforms (right panel) for each spike type. C, Correlation of AP spike type with e-types, represented as the percentage of AP-types associated with each e-type. Download Figure 2-1, TIF file. [file enu-eN-NWR-0510-21-s01.tif]
